# Association of triglyceride-glucose muscle loss index with major adverse cardiovascular events in angina patients: the potential mediating role of inflammation

**DOI:** 10.3389/fmed.2026.1813601

**Published:** 2026-05-29

**Authors:** Yazhao Sun, Xiao Yu, Tianhao Zhang, Chunlan Bai, Lingxiao Zhang, Ya Ma

**Affiliations:** 1 Department of Cardiology, Cangzhou People's Hospital, Cangzhou, Hebei, China; 2 Department of Neurology Intervention, Cangzhou People's Hospital, Cangzhou, Hebei, China; 3 Department of Ultrasound, Cangzhou People's Hospital, Cangzhou, Hebei, China

**Keywords:** major adverse cardiovascular events, mediating effect, muscle loss index, percutaneous coronary intervention, triglyceride-glucose

## Abstract

**Background:**

This study aims to explore the association between triglyceride-glucose muscle loss index (TyG-MLI) and the risk of major adverse cardiovascular events (MACE), as well as the potential mediating role of inflammation.

**Methods:**

The association between TyG-MLI and MACE was analyzed using Cox proportional hazards models, Kaplan–Meier curves, restricted cubic splines (RCS), receiver operating characteristic (ROC) curves, net reclassification improvement (NRI), integrated discrimination improvement (IDI). A sensitivity analysis comparing multiplicative and additive formulations was performed, and subgroup analysis was conducted. Mediation analysis was conducted to explore the potential contribution of neutrophil-to-lymphocyte ratio (NLR) and systemic immune-inflammation index (SII) to this association.

**Results:**

A total of 858 patients with angina who underwent their first percutaneous coronary intervention (PCI) were enrolled in this study. During the 2-year follow-up, 144 cases of MACE were identified. After adjusting for potential confounders, a significant positive association was found between TyG-MLI and MACE (HR 1.203, 95% CI 1.146–1.262). Kaplan–Meier survival analysis revealed that patients in the highest TyG-MLI tertile had the highest risk. RCS analysis showed a linear relationship between TyG-MLI and MACE (*p*-overall < 0.017; *p*-non-linear = 0.219). ROC curve analysis demonstrated that TyG-MLI outperformed both TyG and MLI in diagnostic ability. NRI and IDI analyses confirmed that TyG-MLI significantly improved risk reclassification compared with the TyG alone (IDI = 0.347, NRI = 0.263, both *p* < 0.001). Sensitivity analysis showed that the multiplicative formulation had superior model fit (AIC = 1799.58) and discrimination (C-index = 0.747) compared with the additive index (AIC = 1826.88, C-index = 0.720). Similar results were observed in subgroup analyses. Mediation analysis indicated that NLR and SII partially accounted for the association between TyG-MLI and MACE, although the proportions mediated were modest (approximately 2.4–2.8%).

**Conclusion:**

TyG-MLI is independently associated with the occurrence of MACE in patients with angina undergoing their first PCI. Inflammatory markers accounted for a small proportion of this association, suggesting that other unmeasured pathways may also be involved.

## Introduction

Cardiovascular diseases remain the leading cause of morbidity and mortality worldwide, posing a significant challenge to global health. Metabolic disorders, particularly insulin resistance (IR) and sarcopenia, are recognized as key pathological factors in the development of cardiovascular diseases (1, 2). However, current cardiovascular risk assessment tools primarily focus on traditional metabolic pathways and fail to adequately consider the combined impact of IR and muscle loss on cardiovascular health. Therefore, there is an urgent need to develop novel biomarkers that can more accurately evaluate the cardiovascular risks associated with these metabolic abnormalities.

IR plays a central role in the onset and progression of cardiovascular diseases. It impairs glucose metabolism, leading to hyperglycemia, which in turn causes vascular damage and metabolic dysfunction (3). In this context, the triglyceride-glucose (TyG) index has been proposed and widely validated as an alternative biomarker for IR, and it has been shown to be closely associated with various cardiovascular diseases (4–7). The TyG index and its derived markers have significant value in cardiovascular risk stratification and prognosis prediction. However, existing studies and risk assessment tools primarily focus on metabolic pathways and fail to fully consider the impact of muscle status on cardiovascular health, even though muscle loss may be a crucial factor in adverse cardiovascular outcomes. As the major organ responsible for glucose consumption, skeletal muscle plays a vital role in maintaining systemic metabolic homeostasis, and sarcopenia is characterized by the loss of skeletal muscle mass and function (8). Studies have found that the serum creatinine/cystatin C (Cr/CysC) ratio, as a simplified index, can effectively identify low muscle mass or sarcopenia (9, 10). A retrospective cohort study showed that the Cr/CysC ratio×100 in elderly female patients with community-acquired pneumonia was closely associated with septic shock (11). Research by Liao et al. indicated that a lower Cr/CysC ratio is positively correlated with low muscle mass, and low muscle mass was found to be an independent risk factor for poor prognosis in hypertensive patients (12). A study based on data from the National Health and Nutrition Examination Survey that included 12,914 participants with a median follow-up of 17.9 years found that the Cr/CysC ratio was negatively correlated with all-cause mortality and cardiovascular mortality (13).

It is noteworthy that there is a close interplay between sarcopenia and IR, where the loss of muscle mass reduces glucose consumption, and IR further promotes muscle degeneration. A cross-sectional study involving 4,030 adult participants found that for each 1-unit increase in the TyG index, the likelihood of sarcopenia increased by 31% (14). Another study also showed that elevated TyG index was significantly associated with the prevalence of sarcopenia in the general population, further strengthening the link between IR and muscle loss (15). However, while the TyG index and the Cr/CysC ratio reflect different aspects of IR and muscle loss, respectively, there is currently no single marker that integrates both. As the primary site of insulin-mediated glucose disposal, skeletal muscle loss impairs glucose uptake and exacerbates the metabolic consequences of insulin resistance. This biological interdependence suggests that the cardiovascular risk conferred by the coexistence of insulin resistance and muscle loss may be greater than the sum of their individual contributions. To capture this potential risk amplification, we constructed TyG-MLI as the product of the TyG index and MLI rather than a simple sum. Therefore, we propose the triglyceride-glucose muscle loss index (TyG-MLI) as a novel biomarker aimed at simultaneously capturing the burden of IR and muscle loss. This study aims to explore the association between TyG-MLI and the occurrence of major adverse cardiovascular events (MACE) in patients with angina undergoing percutaneous coronary intervention (PCI), and further investigate the role of inflammation in this mechanism, providing new insights for precise cardiovascular risk prediction.

## Methods and materials

### Population and data collection

This study was approved by the Ethics Committee of Cangzhou People’s Hospital (Approval No: K2025-130-02). Due to its retrospective design, the Ethics Committee waived the requirement for written informed consent. A total of 858 patients with angina who underwent their first PCI at Cangzhou People’s Hospital between January and June 2023 were enrolled. Exclusion criteria included: a history of coronary artery disease; missing data for calculating TyG-MLI or key variables; heart failure, valvular heart disease, pulmonary heart disease, or severe liver or renal insufficiency; acute or chronic infections, immune system disorders, blood diseases, or use of corticosteroids; malignancy; a life expectancy of less than 2 years; and incomplete follow-up data.

Demographic characteristics, medical history, laboratory test results, and medication use were collected and analyzed from the electronic medical records of patients. The TyG index was calculated using the formula: Ln[(TG (mg/dL) × FPG (mg/dL))/2], MLI was calculated as: CysC (mg/L)/Cr (μmol/L), and the TyG-MLI was calculated as: TyG × MLI (16). BMI was calculated as: weight (kg) / height (m)^2^, NLR was calculated as: neutrophil count (10^9^/L) / lymphocyte count (10^9^/L), and SII was calculated as: platelet count (10^9^/L) × neutrophil count (10^9^/L) / lymphocyte count (10^9^/L).

### Definition of outcomes

Follow-up was conducted through telephone calls, in-person outpatient visits, and rehospitalization. MACE events were observed within 24 months following PCI. The MACE outcome was defined as a composite of all-cause mortality, non-fatal myocardial infarction, non-fatal ischemic stroke, transient ischemic attack, stent thrombosis, and target vessel revascularization.

### Statistical analysis

For continuous variables with non-normal distribution, data were presented as median (interquartile range), while normally distributed variables were expressed as mean (standard deviation). Categorical variables were expressed as number (%). Patients with missing data for key variables were excluded from the analysis; therefore, the final analytical dataset contained no missing values for the variables included in the models. Comparisons between the non-MACE and MACE groups were conducted using the *t*-test, Mann–Whitney U test, or chi-square test, as appropriate. Univariate and multivariate Cox proportional hazards regression analyses were used to assess the association between TyG-MLI and MACE, reporting hazard ratios (HRs) and 95% confidence intervals (CIs). The proportional hazards assumption was tested using Schoenfeld residuals, and no significant violation was observed. The cumulative incidence of MACE by TyG-MLI tertiles was calculated and presented using Kaplan–Meier curves, with comparisons made using the Log-Rank test. The dose–response relationship between TyG-MLI and MACE was assessed using restricted cubic spline (RCS) analysis, with knot points placed at the 5th, 35th, 65th, and 95th percentiles of the data. Receiver operating characteristic (ROC) curves were constructed to compare the predictive performance of TyG-MLI, TyG, and MLI for MACE. To further evaluate the incremental prognostic value of TyG-MLI beyond its individual components, net reclassification improvement (NRI) and integrated discrimination improvement (IDI) analyses were performed. Both continuous NRI and IDI were calculated using a bootstrap method with 1,000 replications, comparing nested Cox proportional hazards models. A sensitivity analysis was performed to compare the multiplicative TyG-MLI index with an additive alternative defined as TyG + MLI. Model performance was assessed using the Akaike information criterion (AIC) and Harrell’s C-index. Stratified analyses were performed based on age (<65 and ≥65 years), gender (male and female), BMI (<25 kg/m^2^ and ≥25 kg/m^2^), diabetes (no and yes), and hypertension (no and yes), and interactions between these stratification factors and TyG-MLI were analyzed. Finally, mediation analysis was conducted to explore the potential mediating role of inflammatory markers in the relationship between TyG-MLI and MACE. Average causal mediation effect (ACME) and average direct effect (ADE) were calculated using the bootstrap method. All tests were two-tailed, with statistical significance set at *p* < 0.05. All statistical analyses were performed using R software version 4.4.2 (R Foundation for Statistical Computing, Vienna, Austria).

## Results

### Baseline characteristics

**Figure 1:**
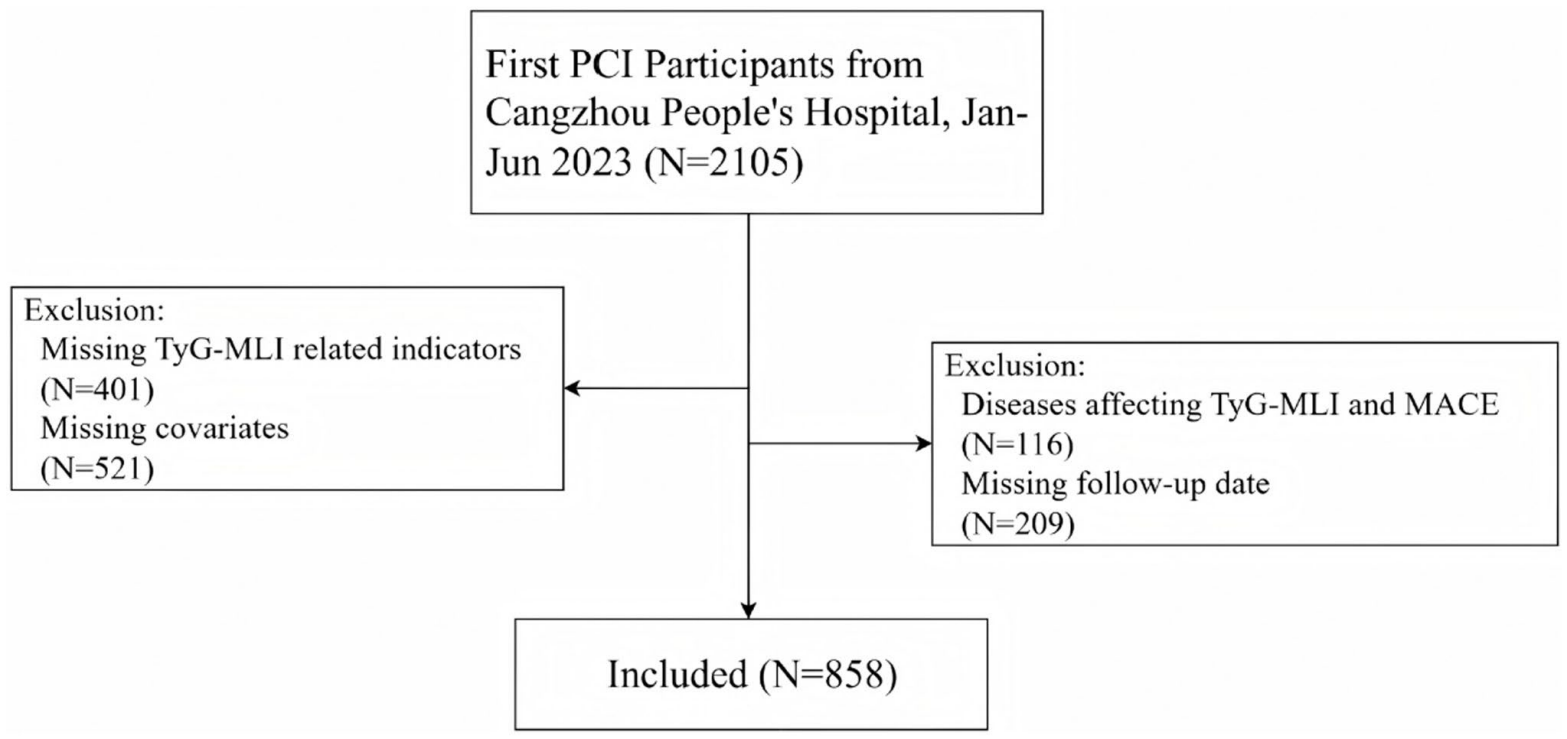
Flow diagram of patient selection. *MACE* major adverse cardiovascular events, *TyG-MLI* triglyceride-glucose muscle-loss index.

A total of 858 participants were ultimately included for analysis. Figure 1 illustrates the screening process for the study population. It is noteworthy that the MACE group exhibited higher levels of age, body mass index, uric acid, total cholesterol, low-density lipoprotein cholesterol, white blood cell, neutrophil, NLR, SII, TyG index, MLI, and TyG-MLI, while showing lower levels of hemoglobin and lymphocyte. Table 1 provides a comprehensive overview of the baseline characteristics.

**Table 1:** Participants demographics and baseline characteristics.

Characteristic	Overall (*N* = 858)	Non-MACE group (*N* = 714)	MACE group (*N* = 144)	*p* value
Clinical variables				
Age, years (continuous)	65.70 ± 6.59	65.36 ± 6.84	67.38 ± 4.82	< 0.001
Age, years (category)				< 0.001
<65	371 (43.2)	327 (45.8)	44 (30.6)	
≥65	487 (56.8)	387 (54.2)	100 (69.4)	
Gender (%)				0.632
Female	328 (38.2)	276 (38.7)	52 (36.1)	
Male	530 (61.8)	438 (61.3)	92 (63.9)	
Current smoking status (%)	193 (22.5)	163 (22.8)	30 (20.8)	0.679
BMI, kg/m^2^ (continuous)	24.48 ± 2.10	24.39 ± 2.03	24.93 ± 2.38	0.012
BMI, kg/m^2^ (category)				0.043
< 25	500 (58.3)	427 (59.8)	73 (50.7)	
≥ 25	358 (41.7)	287 (40.2)	71 (49.3)	
Diabetes (%)				0.821
No	622 (72.5)	516 (72.3)	106 (73.6)	
Yes	236 (27.5)	198 (27.7)	38 (26.4)	
Hypertension (%)				0.177
No	338 (39.4)	289 (40.5)	49 (34)	
Yes	520 (60.6)	425 (59.5)	95 (66)	
Laboratory variables				
UA (μmol/L)	307.00 (255.00–365.00)	300.00 (251.00–357.00)	356.00 (277.50–397.50)	< 0.001
TC (mmol/L)	4.35 (3.70, 5.10)	4.34 (3.68, 5.05)	4.43 (3.86, 5.52)	0.026
TG (mmol/L)	1.25 (0.92, 1.71)	1.23 (0.92, 1.67)	1.35 (0.94, 1.91)	0.109
LDL-C (mmol/L)	2.65 (2.05, 3.28)	2.62 (2.03, 3.25)	2.86 (2.18, 3.49)	0.016
HDL-C (mmol/L)	1.24 (1.03, 1.45)	1.24 (1.03, 1.45)	1.25 (1.01, 1.47)	0.775
WBC (10^9^/L)	6.11 (5.28–7.06)	6.03 (5.22–6.89)	6.51 (5.57–7.71)	< 0.001
HGB (g/L)	139.47 ± 13.56	139.97 ± 13.51	137.01 ± 13.58	0.018
PLT (10^9^/L)	220 (184, 256)	221 (185, 255)	210.5 (182, 257)	0.362
Neu (10^9^/L)	3.98 (3.18–5.09)	3.89 (3.07–4.98)	4.21 (3.60–5.77)	< 0.001
Lym (10^9^/L)	1.43 (1.10–1.83)	1.48 (1.11–1.87)	1.31 (1.03–1.61)	< 0.001
Mono (10^9^/L)	0.36 (0.29–0.48)	0.37 (0.29–0.47)	0.36 (0.28–0.49)	0.930
NLR	2.68 (1.93–4.06)	2.57 (1.86–3.80)	3.39 (2.38–4.88)	< 0.001
SII	595.24 (408.56–923.21)	548.97 (397.76–885.26)	745.76 (537.21–1075.12)	< 0.001
TyG index	8.62 (8.3, 8.95)	8.61 (8.28, 8.93)	8.75 (8.39, 9.01)	0.018
MLI	1.36 (1.13, 1.65)	1.31 (1.1, 1.58)	1.63 (1.35, 1.9)	< 0.001
TyG-MLI	11.66 (9.67, 14.3)	11.31 (9.42, 13.82)	13.63 (11.55, 16.86)	< 0.001
Medications variables				
Aspirin (%)	853 (99.4)	710 (99.4)	143 (99.3)	1.000
Statins (%)	828 (96.5)	689 (96.5)	139 (96.5)	1.000
β-Blocker (%)	452 (52.7)	376 (52.7)	76 (52.8)	1.000
ACEI/ARB (%)	267 (31.1)	224 (31.4)	43 (29.9)	0.796

MACE, major adverse cardiovascular events; BMI, body mass index; UA, uric acid; TC, total cholesterol; TG, triglycerides; LDL-C, low-density lipoprotein cholesterol; HDL-C, high-density lipoprotein cholesterol; WBC, white blood cell; HGB, hemoglobin; PLT, platelet count; Neu, neutrophil; Lym, lymphocyte; Mono, monocyte; NLR, neutrophil-to-lymphocyte ratio; SII, systemic immune-inflammation index; TyG, triglyceride-glucose; MLI, muscle-loss index; TyG-MLI, triglyceride-glucose muscle-loss index; ACEI/ARB, angiotensin-converting enzyme inhibitor/angiotensin II receptor blocker.

### Relationship between TyG-MLI and MACE

**Figure 2:**
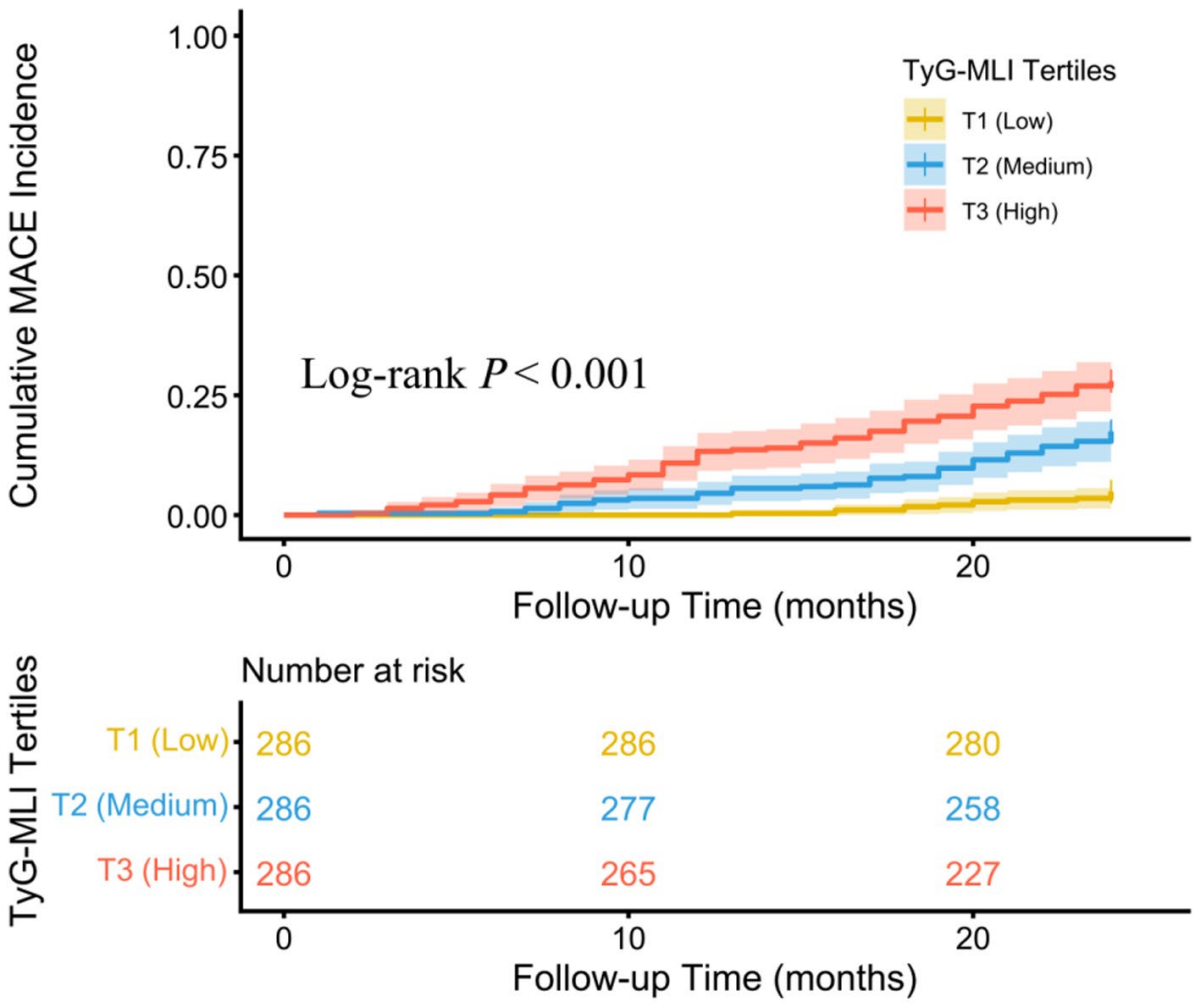
Kaplan–Meier curves of participants according to the tertiles of the TyG-MLI. MACE major adverse cardiovascular events, TyG-MLI triglyceride-glucose muscle-loss index.

**Figure 3:**
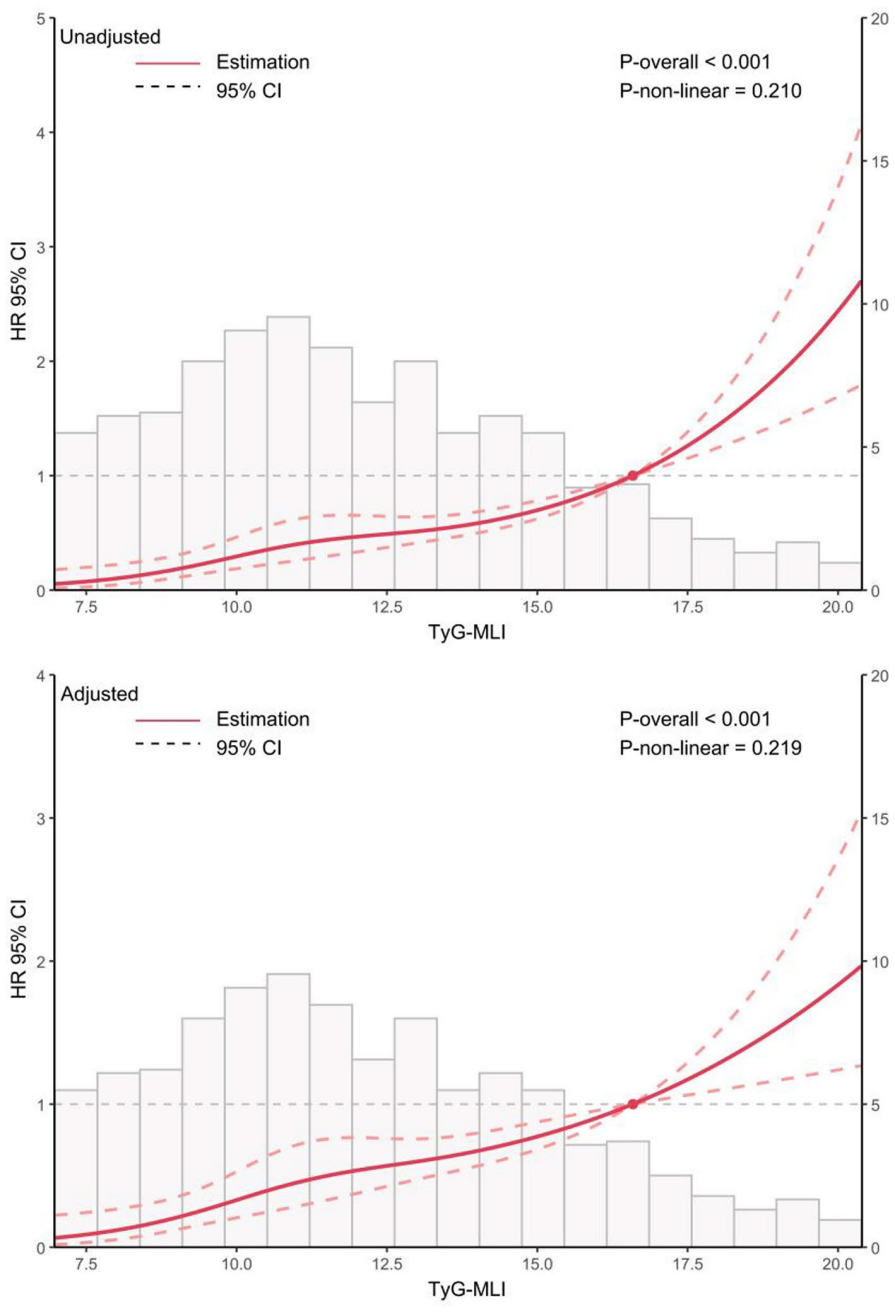
Analysis of RCS. HR hazard ratio, CI confidence interval, TyG-MLI triglyceride-glucose muscle-loss index. No nonlinear relationship between TyG-MLI and the risk of MACE was observed in both the unadjusted and adjusted models. Adjusted for age, body mass index, uric acid, low-density lipoprotein cholesterol, and hemoglobin.

The median follow-up duration in this study was 24 months, during which 144 cases of MACE were recorded. Table 2 presents the results of the Cox regression analysis assessing the association between TyG-MLI and MACE. In the univariate Cox regression model, TyG-MLI was positively associated with the risk of MACE, with a HR of 1.247 (95% CI: 1.190–1.307, *p* < 0.001). The risk of MACE increased progressively from the first to the third tertile of TyG-MLI. After adjusting for multiple potential confounders, the association weakened; however, for each standard deviation increase in TyG-MLI, the risk of MACE increased by 20.3% (HR = 1.203, 95% CI: 1.146–1.262). Compared to the first tertile, the third tertile of TyG-MLI remained positively associated with MACE risk (HR: 6.046, 95% CI: 3.403–10.743). The Kaplan–Meier curve showed a significantly higher cumulative risk of MACE in individuals with higher TyG-MLI (log-rank test, *p* < 0.001) (Figure 2). Furthermore, the RCS analysis revealed a significant linear relationship between TyG-MLI and MACE, which remained stable after adjusting for factors such as age, body mass index, uric acid, low-density lipoprotein cholesterol, and hemoglobin (*p*-non-linearity = 0.219) (Figure 3).

**Table 2:** HRs (95%CIs) for MACE by TyG-MLI.

Variable	Unadjusted			Adjusted		
	HR (95% CI)	*p* value	*p* for trend	HR (95% CI)	*p* value	*p* for trend
TyG-MLI			<0.001			<0.001
Continuous	1.247 (1.190–1.307)	<0.001		1.203 (1.146–1.262)	<0.001	
Categorical						
T1 (Low)	Ref.			Ref.		
T2 (Medium)	3.821 (2.112–6.912)	<0.001		3.622 (1.999–6.562)	<0.001	
T3 (High)	6.741 (3.820–11.896)	<0.001		6.046 (3.403–10.743)	<0.001	

HR, hazard ratio; CI, confidence interval; TyG-MLI, triglyceride-glucose muscle-loss index. Adjusted for age, body mass index, uric acid, low-density lipoprotein cholesterol, and hemoglobin.

### Analysis of the ROC curve

**Figure 4:**
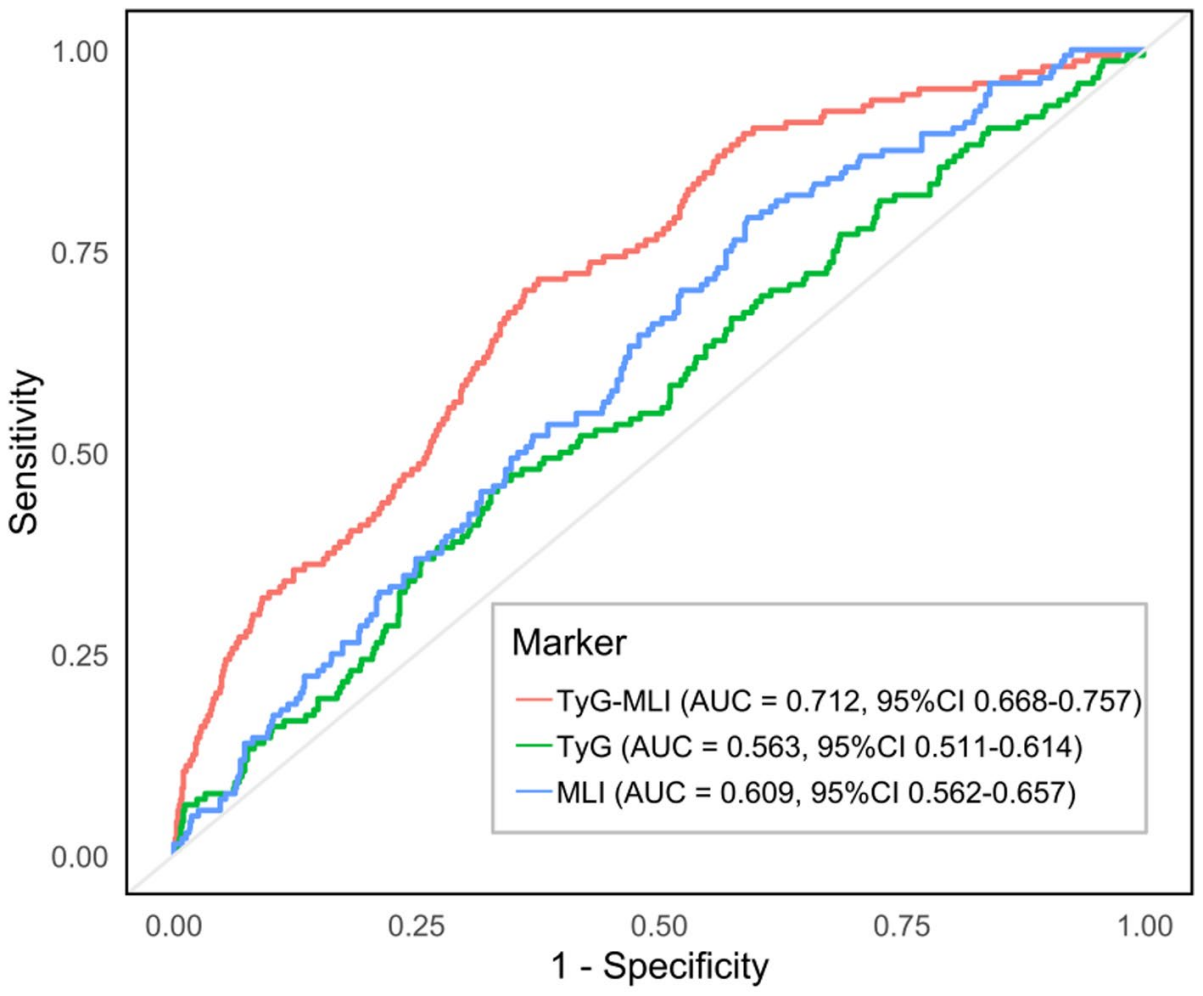
Analysis of the ROC curve. TyG triglyceride-glucose, MLI muscle-loss index, TyG-MLI triglyceride-glucose muscle-loss index.

This study evaluated the ability of TyG, MLI, and TyG-MLI to differentiate outcome events using ROC curve analysis. As shown in Figure 4, the AUC for TyG was 0.563 (95% CI: 0.511–0.614), and the AUC for MLI was 0.609 (95% CI: 0.562–0.657), demonstrating similar predictive capabilities (Z = 1.778, DeLong *p* = 0.075). When TyG and MLI were combined into TyG-MLI, the AUC increased to 0.712 (95% CI: 0.668–0.757), with TyG (Z = 4.707, DeLong *p* < 0.001) and MLI (Z = 2.963, DeLong *p* = 0.003) showing significantly improved predictive performance compared to TyG-MLI.

### Incremental prognostic value of TyG-MLI

To further evaluate whether TyG-MLI provides additional prognostic information beyond its individual components, we performed NRI and IDI analyses. As shown in Table 3, the addition of TyG-MLI to the base model (adjusted for age, body mass index, uric acid, low-density lipoprotein cholesterol, and hemoglobin) significantly improved both discrimination and reclassification, with an IDI of 0.361 (95% CI: 0.259–0.484, *p* < 0.001) and an NRI of 0.269 (95% CI: 0.217–0.298, *p* < 0.001). When TyG-MLI was added to a model already containing the TyG, the IDI was 0.347 (95% CI: 0.248–0.473, *p* < 0.001) and the NRI was 0.263 (95% CI: 0.214–0.295, *p* < 0.001). The addition of TyG-MLI to a model already containing MLI resulted in an IDI of 0.018 (95% CI: −0.004-0.058, *p* = 0.162) and an NRI of 0.014 (95% CI: −0.050-0.079, *p* = 0.608). The addition of the TyG alone to the base model did not significantly improve discrimination or reclassification (IDI = 0.016, *p* = 0.112; NRI = 0.003, *p* = 0.724).

**Table 3:** Incremental prognostic value of TyG-MLI for MACE assessed by IDI and NRI.

Model comparison	IDI (95% CI)	*p* value	NRI (95% CI)	*p* value
Base + TyG vs. Base	0.016 (−0.003–0.047)	0.112	0.003 (−0.042–0.070)	0.724
Base + MLI vs. Base	0.345 (0.242–0.466)	<0.001	0.270 (0.226–0.302)	<0.001
Base + TyG-MLI vs. Base	0.361 (0.259–0.484)	<0.001	0.269 (0.217–0.298)	<0.001
Base + TyG-MLI vs. Base + TyG	0.347 (0.248–0.473)	<0.001	0.263 (0.214–0.295)	<0.001
Base + TyG-MLI vs. Base + MLI	0.018 (−0.004–0.058)	0.162	0.014 (−0.050–0.079)	0.608

IDI, integrated discrimination improvement; NRI, net reclassification improvement; CI, confidence interval; TyG, triglyceride-glucose; MLI, muscle-loss index; TyG-MLI, triglyceride-glucose muscle-loss index. Base model includes age, body mass index, uric acid, low-density lipoprotein cholesterol, and hemoglobin.

### Joint effect assessment and sensitivity analysis

To further evaluate the combined impact of insulin resistance and muscle loss, patients were stratified into four groups according to the median values of TyG and MLI. As shown in Table 4, compared with the Low TyG / Low MLI reference group, the High TyG / Low MLI group had a HR of 1.090 (95% CI: 0.570–2.083, *p* = 0.795), the Low TyG / High MLI group had a HR of 2.796 (95% CI: 1.602–4.878, *p* < 0.001), and the High TyG / High MLI group exhibited the highest risk with a HR of 3.304 (95% CI: 1.911–5.712, *p* < 0.001). In addition, a formal test of multiplicative interaction between TyG and MLI was performed in the fully adjusted Cox model. The interaction term (TyG × MLI) was not statistically significant (*p* = 0.595).

**Table 4:** Joint effect of TyG and MLI on MACE risk.

Group	HR (95% CI)	*p* value
Low TyG/Low MLI	1.000 (Reference)	—
High TyG/Low MLI	1.090 (0.570–2.083)	0.795
Low TyG/High MLI	2.796 (1.602–4.878)	<0.001
High TyG/High MLI	3.304 (1.911–5.712)	<0.001

HR, hazard ratio; CI, confidence interval; TyG, triglyceride-glucose; MLI, muscle-loss index. Adjusted for age, body mass index, uric acid, low-density lipoprotein cholesterol, and hemoglobin.

A sensitivity analysis was performed to compare the multiplicative TyG-MLI index with an additive alternative (TyG + MLI). Model performance was assessed using the AIC and Harrell’s C-index. As shown in Table 5, the multiplicative index yielded a lower AIC (1799.58) and a higher C-index (0.747) than the additive index (AIC = 1826.88, C-index = 0.720) and the base model (AIC = 1852.51, C-index = 0.689), indicating better model fit and discriminative ability for the multiplicative formulation.

**Table 5:** Sensitivity analysis comparing multiplicative and additive formulations of TyG-MLI.

Model	AIC	C-index
Base	1852.51	0.689
Base + TyG-MLI (multiplicative)	1799.58	0.747
Base + TyG + MLI (additive)	1826.88	0.720

AIC, Akaike information criterion; TyG; triglyceride-glucose; MLI, muscle-loss index; TyG-MLI, triglyceride-glucose muscle-loss index. Base model includes age, body mass index, uric acid, low-density lipoprotein cholesterol, and hemoglobin.

### Subgroup analyses

Table 6 presents the results of subgroup analyses examining the relationship between TyG-MLI and MACE across various covariates, including age, Gender, body mass index, diabetes, and hypertension. The association between TyG-MLI and the risk of MACE remained consistently positive across all subgroups. None of these factors significantly modified the relationship between TyG-MLI and MACE.

**Table 6:** Subgroup analysis of the association between TyG-MLI and MACE.

Characteristic	*n* (%)	HR (95% CI)	*p* value	*p* for interaction
Age (years)				0.316
< 65	371 (43.2)	1.159 (1.051–1.278)	0.003	
≥ 65	487 (56.8)	1.215 (1.150–1.284)	<0.001	
Gender				0.830
Female	328 (38.2)	1.209 (1.117–1.309)	<0.001	
Male	530 (61.8)	1.204 (1.132–1.281)	<0.001	
BMI (kg/m^2^)				0.992
< 25	500 (58.3)	1.199 (1.121–1.281)	<0.001	
≥ 25	358 (41.8)	1.220 (1.135–1.311)	<0.001	
Diabetes				0.936
No	622 (72.5)	1.206 (1.137–1.280)	<0.001	
Yes	236 (27.5)	1.215 (1.113–1.327)	<0.001	
Hypertension				0.782
No	338 (39.4)	1.200 (1.111–1.297)	<0.001	
Yes	520 (60.6)	1.205 (1.133–1.282)	<0.001	

HR, hazard ratio; CI, confidence interval; BMI, body mass index. Adjusted for age, body mass index, uric acid, low-density lipoprotein cholesterol, and hemoglobin.

### Mediation analysis

This study also explored whether inflammatory indicators contribute to the association between TyG-MLI and MACE. The Bootstrap mediation analysis results presented in Table 7 indicated that NLR and SII partially accounted for the association between TyG-MLI and MACE, with modest mediation proportions of approximately 2.8 and 2.4%, respectively. Given the small magnitude of these effects, the clinical relevance of inflammation as a mediator should be interpreted with caution.

**Table 7:** Mediation analysis assessing the contribution of inflammatory indicators to the association between TyG-MLI and MACE.

Variable	Path	Unstd. effect	SE	95%CI	Std. Prop.
NLR	ACME	0.005	0.003	0.002–0.012	0.028
	ADE	0.182	0.026	0.134–0.237	-
	Total	0.187	0.026	0.139–0.243	-
SII	ACME	0.004	0.002	0.001–0.010	0.024
	ADE	0.181	0.026	0.134–0.236	-
	Total	0.185	0.026	0.138–0.242	-

Unstd., effect unstandardized effect; SE, standard error; CI, confidence interval; Std. Prop., standardized effect proportion; NLR, neutrophil-to-lymphocyte ratio; SII, systemic immune-inflammation index; ACME, average causal mediation effect; ADE, average direct effect. Adjusted for age, body mass index, uric acid, low-density lipoprotein cholesterol, and hemoglobin.

## Discussion

This study introduces TyG-MLI as an innovative biomarker that integrates the dual impacts of IR and muscle loss, offering a novel perspective for predicting MACE. The main findings from our retrospective cohort analysis are summarized as follows: (1) TyG-MLI is an independent predictor of MACE after PCI in patients with angina, with higher values significantly associated with poorer outcomes (HR = 1.203, 95% CI: 1.146–1.262). (2) ROC curve analysis demonstrates that TyG-MLI outperforms both TyG index and MLI in distinguishing MACE. (3) NRI and IDI analyses confirm that TyG-MLI provides significant incremental prognostic value beyond the TyG index alone (IDI = 0.347, NRI = 0.263, both *p* < 0.001), primarily by improving risk reclassification. (4) Sensitivity analysis further supports the multiplicative formulation over an additive alternative in terms of model fit and discrimination. (5) Elevated inflammatory markers, such as NLR and SII, accounted for a small proportion of the association between TyG-MLI and MACE.

The TyG-MLI, as a novel composite biomarker that integrates the risks of IR and skeletal muscle loss, holds potential value in cardiovascular risk assessment. A cross-sectional study involving 7,161 participants aged 18 and older from the National Health and Nutrition Examination Survey demonstrated a positive correlation between the TyG index and its derived markers with the risk of sarcopenia (17), which is consistent with the theoretical basis of the TyG-MLI index that combines IR and muscle status. A large-scale prospective study based on the UK Biobank, which included hundreds of thousands of adult participants, found that TyG-MLI levels were significantly positively correlated with the overall risk of cardiovascular disease. Moreover, even in populations with normal or low body weight, this association remained significantly stronger than the TyG alone. This suggests that TyG-MLI is capable of capturing latent cardiovascular metabolic risks that are not reflected by traditional metabolic risk indicators (16).

To further quantify the incremental prognostic value of TyG-MLI beyond its individual components, we performed NRI and IDI analyses. Notably, the addition of TyG-MLI to a model already containing the TyG yielded an IDI increase of 34.7% and an NRI increase of 26.3%. This indicates that TyG-MLI successfully identifies a subset of high-risk patients who would be misclassified by conventional insulin resistance assessment alone, specifically those with relatively normal glycometabolic profiles yet significant muscle loss. Conversely, the incremental benefit of TyG-MLI over MLI alone was not statistically significant, suggesting that muscle loss itself is the predominant driver of adverse outcomes, while TyG-MLI serves to amplify risk signals by integrating both metabolic and muscular dimensions. To provide statistical justification for the multiplicative formulation of TyG-MLI, we conducted a sensitivity analysis comparing the multiplicative index with a simple additive alternative (TyG + MLI). The multiplicative form demonstrated superior model fit and discriminative performance, with a lower AIC (1799.58 vs. 1826.88) and a higher C-index (0.747 vs. 0.720). This finding aligns with the approach of previously established composite indices in cardiovascular research, such as the TyG-body mass index and TyG-waist circumference index, both of which adopt a multiplicative structure to capture the interplay between metabolic and anthropometric risk factors (18, 19). The consistency across these indices supports the rationale that multiplying two interrelated risk dimensions provides a more sensitive marker of adverse outcomes than simple addition.

IR affects the cardiovascular system through multiple pathophysiological pathways and plays a key role in the development of atherosclerosis and MACE. Insulin signaling dysfunction leads to abnormal glucose metabolism and endothelial dysfunction, accelerating the progression of atherosclerosis while promoting a hypercoagulable state and inflammatory response. These mechanisms are closely associated with cardiovascular events such as coronary artery disease (20). Chronic low-grade inflammation is a significant concomitant of IR, which disrupts endothelial cells’ normal response to insulin and further exacerbates endothelial damage and thrombosis through the release of inflammatory mediators, thereby increasing the risk of MACE (21). Furthermore, IR can activate the sympathetic nervous system, elevate blood pressure, and cause lipid metabolism disturbances that lead to dyslipidemia. These changes collectively worsen arteriosclerosis and cardiovascular burden (22, 23). Meanwhile, the decline in muscle mass leads to glucose metabolism abnormalities, increased fat accumulation, and activation of chronic inflammation, thereby exacerbating systemic metabolic imbalance and endothelial dysfunction. These changes are closely related to the elevated risk of cardiovascular and cerebrovascular events (24, 25). Relevant studies suggest that sarcopenia may promote atherosclerosis and other cardiovascular diseases through common mechanisms such as pro-inflammatory responses, oxidative stress, and IR, thereby forming a vicious cycle (26–28). In addition, the inflammatory and metabolic disorders commonly observed in sarcopenia are considered key factors in exacerbating cardiovascular risk, providing mechanistic support for the relationship between muscle mass loss and cardiovascular events.

This study also explored whether inflammatory markers contribute to the association between TyG-MLI and MACE. Mediation analysis indicated that NLR and SII accounted for approximately 2.8 and 2.4% of the total association, respectively. Although statistically significant, these proportions are small, and therefore the clinical relevance of inflammation as an explanatory pathway should be interpreted with caution. These findings suggest that inflammation represents only one of several potential downstream factors linking insulin resistance and muscle loss to adverse cardiovascular outcomes, while the majority of the association is likely attributable to other unmeasured mechanisms. To further clarify the mode of contribution of TyG and MLI, a multiplicative interaction test was performed. The interaction term (TyG × MLI) was not statistically significant (*p* = 0.595), indicating the absence of synergistic or antagonistic interaction on a multiplicative scale. Given the distinct etiological origins of insulin resistance and muscle loss, together with the modest proportion of the combined effect mediated by inflammation, these two factors likely contribute to MACE through partially overlapping yet relatively independent pathways. Accordingly, the clinical utility of TyG-MLI as a multiplicative composite index lies primarily in its ability to amplify prognostic signals by integrating two distinct dimensions of risk, rather than in capturing a biological synergistic effect.

Although this study provides preliminary evidence for the potential of TyG-MLI in predicting MACE risk, several limitations should be noted. First, this is a single-center retrospective cohort analysis with a relatively short recruitment period (January to June 2023), and due to its design, it is difficult to make causal inferences, and there may be selection bias, which could limit the generalizability of the conclusions. Second, while TyG-MLI can comprehensively consider the dual impact of insulin resistance and muscle loss, the generalizability of this index has not been validated in independent external cohorts. Third, the assessment of sarcopenia in this study relied solely on laboratory measures of CysC and Cr as a surrogate index, without incorporating imaging-based assessments (e.g., dual-energy X-ray absorptiometry or computed tomography) or measurements of muscle strength and physical performance. This limits the study’s ability to capture the multidimensional characteristics of sarcopenia as defined by current consensus guidelines. Fourth, although sensitivity analysis supported the multiplicative over the additive formulation, the optimal mathematical form of TyG-MLI, such as whether weighted coefficients should be introduced, remains to be validated in external cohorts. Fifth, although patients with severe renal insufficiency were excluded and no significant differences in baseline medication use were observed between groups, residual confounding from unmeasured factors, such as disease severity (including coronary artery disease burden), medication adherence, and baseline inflammatory status, cannot be entirely ruled out. Future studies incorporating more comprehensive clinical and angiographic data are warranted to validate our findings. Sixth, the predictive performance of TyG-MLI, although superior to its individual components, remained moderate, and direct comparison with established risk scores was not performed due to the retrospective nature of the study and limited availability of required variables. Future prospective studies incorporating head-to-head comparisons with existing risk stratification tools are warranted to further define the incremental clinical utility of TyG-MLI. Finally, while inflammation may play a contributory role in the association between TyG-MLI and MACE, the specific pathways involved and the interactions with other potential biomarkers, such as high-sensitivity C-reactive protein, still require further investigation. Future prospective multicenter studies will help to further evaluate the application value of TyG-MLI in diverse clinical populations and clarify its potential role in cardiovascular risk assessment.

## Conclusion

In conclusion, TyG-MLI has been demonstrated to be a more effective predictor of MACE compared to the use of TyG or MLI alone. Inflammatory markers accounted for a small proportion of this association, highlighting the need for further investigation into other potential underlying mechanisms.

## Data Availability

The original contributions presented in the study are included in the article/supplementary material, further inquiries can be directed to the corresponding author.

## Glossary

ACEI/ARB: Angiotensin-converting enzyme inhibitor/angiotensin II receptor blocker
ACME: Average causal mediation effect
ADE: Average direct effect
AIC: Akaike information criterion
AUC: Area under the curve
BMI: Body mass index
CI: Confidence interval
Cr/CysC: Creatinine/cystatin C
HDL-C: High-density lipoprotein cholesterol
HGB: Hemoglobin
HR: Hazard ratio
IDI: Integrated discrimination improvement
IR: Insulin resistance
LDL-C: Low-density lipoprotein cholesterol
Lym: Lymphocyte
MACE: Major adverse cardiovascular events
MLI: Muscle loss index
Mono: Monocyte
Neu: Neutrophil
NLR: Neutrophil-to-lymphocyte ratio
NRI: net Reclassification improvement
PCI: Percutaneous coronary intervention
PLT: Platelet count
RCS: Restricted cubic spline
ROC: Receiver operating characteristic
SE: Standard error
SII: Systemic immune-inflammation index
Std: Prop. standardized effect proportion
TC: Total cholesterol
TG: Triglycerides
TyG: Triglyceride-glucose
TyG-MLI: Triglyceride-glucose muscle loss index
UA: Uric acid
Unstd: Effect unstandardized effect
WBC: White blood cell
